# High-dose chemotherapy for patients with stage III breast cancer with homologous recombination deficiency: a discrete choice experiment among healthcare providers

**DOI:** 10.2340/1651-226X.2024.40276

**Published:** 2024-09-10

**Authors:** Joost G.E. Verbeek, Leyla Azarang, Luis E. Pilli, Vincent M.T. de Jong, Agnes Jager, Sabine C. Linn, Valesca P. Retèl, Wim H. van Harten

**Affiliations:** a Division of Psychosocial Research and Epidemiology, The Netherlands Cancer Institute, Amsterdam, The Netherlands; b Department of Health Technology and Services Research, University of Twente, Enschede, The Netherlands; c Erasmus School of Health Policy & Management, Erasmus University, Rotterdam, the Netherlands; d Erasmus Choice Modelling Centre, Erasmus University, Rotterdam, the Netherlands; e Erasmus Centre for Health Economics Rotterdam, Erasmus University, Rotterdam, the Netherlands; f Department of Molecular Pathology, Antoni van Leeuwenhoek Hospital – Netherlands Cancer Institute, Amsterdam, The Netherlands; g Department of Medical Oncology, Erasmus MC Cancer Institute, Rotterdam, The Netherlands; h Department of Medical Oncology, Antoni van Leeuwenhoek Hospital – Netherlands Cancer Institute, Amsterdam, The Netherlands; i Department of Pathology, Utrecht University Medical Center, Utrecht, The Netherlands

**Keywords:** Breast neoplasms, homologous recombination, BRCA1 protein, BRCA2 protein, discrete choice experiment, conjoint analysis, implementation science

## Abstract

**Background and purpose:**

High-dose chemotherapy with autologous stem cell rescue (HDCT) is currently under investigation as a potential therapy for patients with stage III HER2-negative breast cancer with homologous recombination deficiency (HRD). In addition to survival, the impact on short- and long-term side effects might influence the uptake of HDCT by healthcare professionals. As part of the SUBITO trial, we investigated healthcare professionals’ treatment (outcome) preferences for patients with HRD stage III HER2-negative breast cancer and established how healthcare professionals make trade-offs between these treatment outcomes.

**Patients/material and methods:**

We conducted a discrete choice experiment in which healthcare professionals were asked to choose repeatedly between scenarios with two treatment options (HDCT or standard of care (SOC)) that varied in outcome with respect to 10-year overall survival (OS), short-term toxicity, long-term cognitive impairment, and premature menopause. We analysed treatment preferences, relative importance, and trade-offs using a multinomial logistic model.

**Results and interpretation:**

Thirty-five of the 151 dedicated breast cancer professionals with extensive experience in treating breast cancer patients completed the survey. The 10-year OS and long-term cognitive impairment were the most important attributes. The results indicate a requirement of 10.4% and 25.1% absolute additional improvement in the 10-year survival rate to justify accepting moderate or severe long-term cognitive impairment as a trade-off, respectively. Therefore, we found in our dataset that healthcare professionals expected a large improvement in 10-year OS to accept moderate to severe cognitive impairment. This information calls for further research into chemotherapy-related cognitive impairment, shared decision-making, and treatment preferences for patients with stage III breast cancer.

## Introduction

High-dose chemotherapy with autologous stem cell rescue (HDCT) is a potential treatment for stage III HER2-negative breast cancer patients with homologous recombination-deficient (HRD) tumours [1–3]. Unplanned retrospective analysis revealed an absolute improvement from 30% to 78% in 7-year recurrence-free survival for HDCT compared with conventional chemotherapy (hazard ratio 0.12 (95% CI: 0.04–0.43)) in patients with HRD tumours [1]. This finding is in line with other findings suggesting that alkylating chemotherapy is beneficial for patients with HRD tumours [4]. To confirm these results and investigate short- and long-term side effects, cost-effectiveness, and quality of life, an international multicentre randomised controlled trial was initiated (SUBITO trial; NCT02810743) in which patients with stage III HER2-negative breast cancer were randomised to either HDCT or the Dutch standard of care plus a poly (ADP-ribose) polymerase (PARP) inhibitor (i.e. olaparib). The study commenced on January 25, 2017, with an estimated primary completion date of October 1, 2024, and an anticipated overall study completion date of December 1, 2033 [5].

The SUBITO trial is part of a coverage with evidence development trajectory, meaning that the results of the study will be followed by a swift reimbursement decision [6]. In this process, it is important to identify factors that affect the uptake of a health technology into routine use, as this is a social process with multiple determinants that go well beyond the evidence supporting the innovation itself [7–10]. One strategy to systematically evaluate treatment preferences and engage stakeholders in the process is the use of preference elicitation studies such as discrete choice experiments (DCEs), which are increasingly used in the healthcare sector [11].

DCEs are survey-based experiments that contribute to informed decision-making in several ways, such as the elicitation of treatment preferences and trade-offs between treatment characteristics, the prediction of the uptake of a new health technology, and the identification of relevant outcomes for economic evaluations [12–15]. In combination with qualitative research on implementation factors, these factors can be used to inform the design of health technologies and identify potential barriers and facilitators of uptake [12–15]. Furthermore, DCEs help with uncovering the decision process, which in turn may lead to the development of better shared decision-making strategies between patients and physicians [16]. Shared decision-making techniques might be used by healthcare professionals to minimise patients’ post-treatment decisional regret, particularly when patients’ and healthcare providers’ views and beliefs are not the same [17, 18].

In a recently conducted qualitative study, it was observed that the uptake of HDCT by healthcare professionals might be influenced by concerns about potential toxicity and late effects of the treatment [19]. Moreover, the respondents in this study mentioned that the complexity of the treatment steps, the potential pre-existing treatment preference of healthcare providers, and the current patient information materials could be issues for the uptake and high-quality deliberation and communication of treatment plans [19]. To understand these hurdles for implementation and uptake, we aimed to evaluate healthcare professionals’ treatment (outcome) preferences for patients with HRD stage III HER2-negative breast cancer and establish how healthcare professionals make trade-offs between these treatment outcomes.

## Materials and methods

### Discrete choice experiment

In the current DCE, healthcare providers were asked to choose between HDCT and standard-dose chemotherapy with olaparib, with different hypothetical outcomes (i.e. levels of the attributes). The healthcare professionals were asked to make a series of fifteen hypothetical choices. We followed the DCE guidelines of the Professional Society for Health Economics and Outcomes Research (ISPOR) to determine the attributes and their levels, the experimental design, and the statistical analysis [12, 20, 21]. A detailed written explanatory statement was given to respondents describing the study, which highlighted that their participation was voluntary, that no identifiable personal data would be collected, and that responses would be used for research purposes.

### Treatment options: Standard-dose chemotherapy with olaparib and HDCT

In the questionnaire, we outlined two treatment options and asked the participants to choose between them using hypothetical outcomes. HDCT was described as follows: “Duration of treatment is 16 weeks; four cycles of (neo)adjuvant ‘dose-dense’ doxorubicin + cyclophosphamide, the fourth with stem cell mobilization followed by stem cell harvesting, followed by two cycles of intensified alkylating chemotherapy consisting of cyclophosphamide, thiotepa and carboplatin, completed with stem cell restitution.” For standard-dose chemotherapy with olaparib, the explanation was: “Duration of treatment is 72 weeks; four cycles of (neo)adjuvant dose-dense doxorubicin + cyclophosphamide, followed by four cycles of three-weekly carboplatin combined with weekly paclitaxel, followed by one year of adjuvant olaparib.”

### Attributes and levels

To identify DCE attributes, we used exploratory semi-structured interviews. Full details on the methods and results of the interviews are provided elsewhere [19]. A total of four attributes were selected by the study team [22]. For this selection, we conducted a ranking exercise with three medical oncologists and two senior researchers to determine the attributes and their levels. Specifically, we showed the experts thirteen potential attributes identified from the qualitative research and asked them to rank them from most relevant to least relevant treatment attribute (see Supplementary Appendix A). The ranking exercise and subsequent open discussions with the experts revealed that four attributes were consistently perceived as important: 10-year overall survival (OS) (50%, 60%, 70%, 80%, 90%), short-term treatment toxicity (grade 2, grade 3, grade 4), long-term cognitive impairment (mild, moderate, severe), and premature menopause (10–30%, 45–65%, 80–100%). The levels assigned to these attributes were based on the current literature and verified for plausibility by the three medical oncologists on our study team [23–27].

### Experimental design and questionnaire

It is possible to draw 135 possible unique combinations from the four attributes. Therefore, an optimal subset was determined using experimental design techniques to make it feasible for respondents. We used the R package AlgDesign version 1.2.0. to select the subset that maximises the D-efficiency (i.e. minimises the generalised variance of the parameter estimates) for a given number of choice sets [28]. Moreover, we restricted the experimental design, as suggested by clinical experts, removing all overlapping, dominant, and implausible choice sets, that is, combinations in which the chance of premature menopause was lower within the HDCT treatment compared to standard dosage. In the end, we generated an optimal design with 30 choice sets, divided into two versions of the DCE consisting of fifteen choice sets each. The potential participants were randomised between the two questionnaires (version A or B).

**Figure 1:**
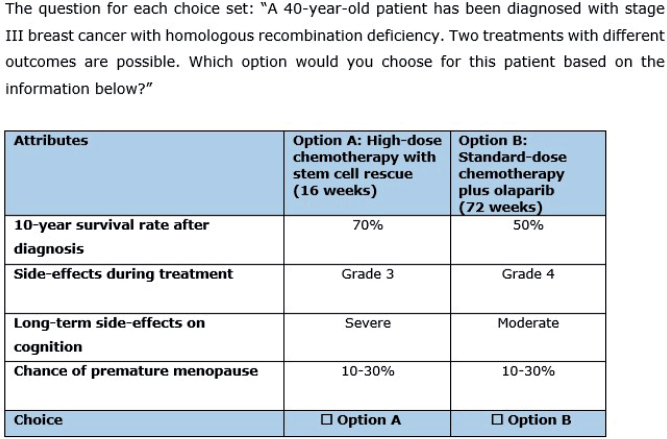
Example of a choice set.

In the questionnaire, demographic questions, such as age, sex, occupation, and experience with treating stage III breast cancer patients, were included. Additionally, to classify the respondents into two groups differing in openness to adaptation, we asked them three questions on innovativeness based on Rogers’ diffusion of innovation curve [9]. Subsequently, a clear description of the attributes, their levels, and an example of a completed choice set were given. Furthermore, participants were informed that the scenarios presented in the questionnaire were hypothetical. An example of a choice set can be found in Figure 1. Finally, an open-ended question was posed asking for missing attributes in the DCE. The questionnaire was only provided in Dutch and can be found along with a translation in Supplementary Appendix B. We did not provide monetary incentives to complete the survey.

The questionnaire was validated for feasibility (i.e. response fatigue) and clarity by an external oncologist (*n* = 1), an oncology nurse practitioner (*n* = 1) and members of the research team (*n* = 5). Comments were addressed accordingly.

### Study population and recruitment

The respondents in this study were recruited from a contact list of oncology healthcare workers in the Netherlands, including 86 medical oncologists, 48 surgical oncologists, and 17 oncology nurse practitioners working in more than 60 different hospitals in the Netherlands.

Because our target population comprises a small population of healthcare professionals, we formulated a comprehensive strategy to enhance the quantity of questionnaire responses. This approach involved the creation of both paper and online survey versions, making personal contact with potential participants, and sending four reminders over an extended period. We concluded the data collection process when no additional responses were received.

### Data analysis

Responses from the healthcare providers were analysed using random utility theory [29, 30]. According to this theory, a multinomial logistic model is used to predict respondents’ choice of treatment as a function of treatment attributes and levels [30]. For this DCE, the utility that a healthcare provider “*n”* assigns to treatment “*j*” in choice task *“t”*, say *U_njt_*, is modelled as the sum of two parts: a systematic part based on the attributes included in the DCE and an error part:


$$U_{njt}=V_{njt}+\varepsilon_{njt}$$

In the expression above, *V_njt_* is the systematic utility of alternative *j* encountered by individual *n* in the choice task *t,* and ε*_j_* represents unmeasured variation in preferences due to individual differences, incomprehensible qualities of treatment, and measurement errors.


$$V_{njt}=ASC_{s}+\sum_{K}\beta_{k}X_{nkjt}$$

In the expression above, *ASC* and *β* are vectors of parameters to be estimated. *ASC_s_* are treatment-specific constants capturing the value of the idiosyncratic qualities of treatment *s, β_k_* are *K* taste parameters that weight attributes to value alternative *j*, and *X_nkjt_* is a vector of *K* attributes describing the alternative *j* encountered by individual n during choice task *t*. We dummy-coded all attributes, except the willingness to survive, which was parametrised as linear. To convert overall survival from a categorical variable to a continuous variable, we conducted a test for linearity. This analysis involved examining the relationship across levels of overall survival, ranging from 40% to 90% chance of 10-year survival, using a model with only categorical variables. Our analysis confirmed that overall survival exhibits linearity among its levels.

Different hypotheses on the distribution of the error, ε, result in different random utility models. Assuming that the ε values are identically independent and follow a Gumbel distribution leads to the following multinomial logit model:


$$P_{\left(choice\;A\right)}=\frac{exp\left(V_{A}\right)}{\exp\;(V_{A})\;+\;\exp\left(V_{B}\right)}$$

The model described was coded in Python, and the parameters were estimated using maximum likelihood. For statistical identification, the parameters for the specific constant for standard of care (SOC) and the betas for grade 2 toxicity, mild long-term side effects on cognition, and a 10–30% likelihood of premature menopause were constrained to zero, that is, defined as reference levels.

The presentation of the alternatives indicates and describes two different treatments (HDCT and SOC) that hold value apart from the specified attributes. As a result, the model considers the different treatment options as variables, following the ISPOR guidelines for Good Practices [31, 32]. The sign of the coefficient (plus or minus) reflects whether a specific attribute has a positive or negative effect on the utility of the treatment compared with the reference level. *P*-values < 0.05 were considered to indicate statistical significance.

Each value of the coefficient indicates the relative contribution of the corresponding attribute level to the utility of the alternative. We calculated the attribute-specific relative importance as a percentage by calculating the difference in utility between the highest and lowest levels within a single attribute divided by the sum of the values of all attributes. For 10-year overall survival (continuous variable), we used a 40% change in attribute level, that is, ranging from 50 to 90%.

Next, we estimated the marginal willingness to trade off treatments and changes in the attributes’ levels by changes in the 10-year OS. In other words, we transformed the model into a 10-year OS framework through the following parametrisation:


$$WTS_{jnt}=-b_{OS}*ASC_{s}+\sum_{K}\beta_{k}X_{kjnt}-OS_{jnt}$$

Here, *WTS_jnt_* denotes the willingness to survive, and the components include alternative-specific constants (treatments), coefficients (*β_k_*, where k represents each attribute, except for the 10-year OS rate), and the level of overall survival (*OS_jnt_*). The model is rescaled by the parameter bOS, enabling the coefficients to be interpreted as the willingness to trade changes in attribute levels for a one percent increase in the overall survival rate. For example, this rescaling allows us to quantify how many percentage points of survival rate a respondent is willing to trade off to accept a higher risk of cognitive impairment, such as changing from moderate to severe.

## Results

### Characteristics of the healthcare professionals

From the 151 questionnaires distributed to healthcare professionals, 35 questionnaires were returned (response rate 23.2%). The healthcare professionals’ characteristics can be found in Table 1. The respondents were medical oncologists (*N* = 24, 68.6%), surgical oncologists (*N* = 8, 22.9%), and oncology nurse practitioners (*N* = 3, 8.6%), with an average of 16 years of experience in treating stage III breast cancer patients. The mean age was 51 years (range: 35–67), the majority were female (*N* = 23, 65.7%), and 19 patients were treated for stage III breast cancer per year on average. Furthermore, most respondents currently assessed HDCT as experimental (*N* = 13, 37.1%), used literature as a source of information to assess the status of HDCT (*N* = 27, 77.1%), and indicated that they had been educated on HDCT (*N* = 23, 65.7%). Six of the 24 medical oncologists were ultimately responsible for treating breast cancer patients with HDCT. Moreover, we rated the respondents based on their innovativeness score as 60% belonging to the majority group and 40% as innovators. Lastly, respondents mentioned that other long-term side effects of the treatment, such as neurotoxicity and chronic fatigue (*n* = 5), being able to return to work (*n* = 4), death due to treatment (*n* = 2), and cardiac side effects (*n* = 2) were also important when making a treatment decision. The other respondents (*n* = 22) did not report any missing attributes.

**Table 1:** Characteristics of the healthcare professionals (*n* = 35).

Age (years, mean ± standard deviation)	51 ± 8.9
Female	23 (65.7%)
Profession	
Medical oncologist	24 (68.6%)
Surgical oncologist	8 (22.9%)
Specialist breast care nurse	3 (8.6%)
Type of setting	
Academic hospital	2 (5.7%)
Comprehensive Cancer Centre	5 (14.3%)
Regional hospital with training	8 (22.9%)
Regional hospital without training	8 (20.0%
STZ-hospital^a^	12 (37.1%)
Years active in the field of breast cancer (years, mean ± standard deviation)	16 ± 7.8
Number of stage III patients under treatment per year (# patients, mean ± standard deviation)	19 ± 13.1
Do you have experience with stem cell rescue?	
Yes	13 (37.1%)
No	22 (62.9%)
Have you been educated on HDCT?	
Yes	23 (65.7%)
No	12 (34.3%)
Have you performed HDCT as a registered, ultimately responsible internist?^b^	
Yes	6 (25.0%)
No	18 (75.0%)
How do you currently classify HDCT?^c^	
Insufficient evidence	7 (20.0%)
Sufficient evidence	4 (11.4%)
Experimental	13 (37.1%)
Other	11 (31.4%)
Which sources do you use to assess the status of HDCT?^c^	
Literature	27 (77.1%)
Field of profession	15 (42.8%)
Experience	5 (14.3%)
Prominent figures	9 (25.7%)
Other	0 (0.0%)
Innovativeness score	
Majority	60%
Innovators	40%

a STZ-hospitals are accredited top clinical hospitals.

b This question was only relevant for medical oncologists (*n* = 24).

c Respondents were able to fill out multiple answers.

HDCT: High-Dose Chemotherapy with Autologous Stem Cell Rescue.

### Preferences of healthcare providers

**Figure 2:**
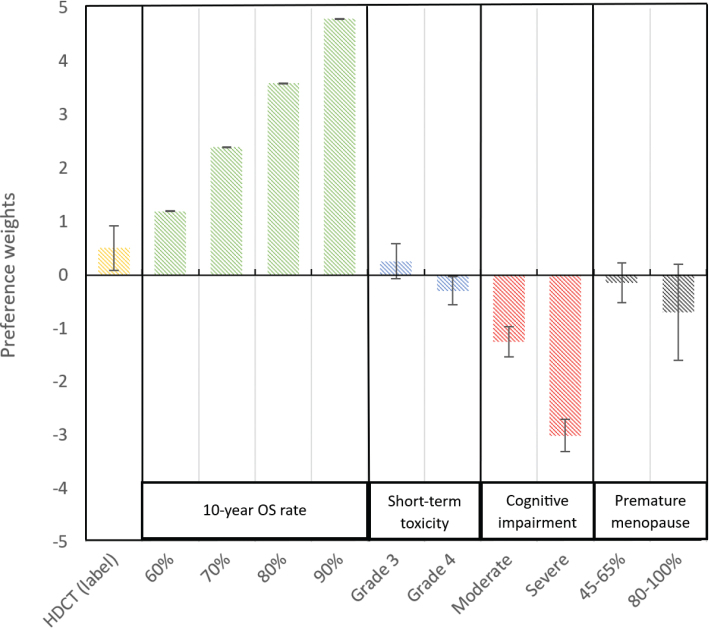
Preference weights of all attribute levels with ±95% confidence intervals, compared to the reference level.

The choice sets scored by the healthcare providers can be found in Supplementary Appendix C, and the results of the multinomial model are displayed in Table 2. For healthcare providers, an improved 10-year OS rate was most important, with a utility of 0.12 (95% CI: 0.11 to 0.13) (*P* < 0.001) per-unit change (on a scale of 50% to 90%). Thus, the utility is 0.12 for a 1% improvement in the 10-year OS. The second most important attribute was long-term cognitive impairment, with a disutility of −1.25 for moderate long-term cognitive impairment (95% CI: −1.81 to −0.69) (*P* < 0.002) and −3.00 for severe long-term cognitive impairment (95% CI: −3.59 to −2.41) (*P* < 0.001) compared to the reference level, that is, mild long-term cognitive impairment. Notably, neither premature menopause nor short-term treatment toxicity had a significant impact on treatment preference. Furthermore, we found no significant difference between the alternative-specific constants of the treatment options (i.e. high-dose chemotherapy versus standard-dose chemotherapy). Figure 2 shows our findings.

**Table 2:** Results from the multinomial model and relative importance of attributes.

Attributes and levels	Estimate	*P*-value	Standard error	Relative importance†
High-dose chemotherapy with stem cell rescue	0.51	0.24	0.41	3.0%
10-year overall survival*	0.12	<0.001	<0.00	60.8%
Short-term treatment toxicity**				7.0%
Grade 3	0.26	0.44	0.33	
Grade 4	−0.29	0.30	0.27	
Long-term cognitive impairment**				22.2%
Moderate	−1.25	0.002	0.28	
Severe	−3.00	<0.001	0.30	
Premature menopause**				7.0%
45–65%	−0.14	0.71	0.38	
80–100%	−0.69	0.45	0.90	

* This attribute is continuous, that is, estimate of one-unit change (1%).

** These attributes are categorical and compared to the reference level (dummy coding), that is, standard-dose chemotherapy plus olaparib, grade 2 toxicity, mild cognitive impairment, 10–30% chance on premature menopause.

† Relative importance in percentage by calculating the difference in utility between the highest and lowest level within a single attribute, divided by the sum of the value of all attributes. For 10-year overall survival (continuous variable), we used a 40% change in attribute level, that is, ranging from 50 to 90%.

The conditional relative importance of the attributes 10-year OS (60.8%) and long-term cognitive impairment (22.2%) was considerably greater than those of premature menopause (7.0%) and short-term treatment toxicity (7.0%), which indicates that 10-year OS and long-term cognitive impairment were deemed more important factors for decision making by healthcare providers. Moreover, the description (i.e. ‘label’) of the treatment option had relatively little effect on the treatment choices of the healthcare providers (3.0%). The preference weights with standard errors of all attribute levels compared to the reference are displayed in Figure 2.

### Willingness to trade attributes

To make the results of the multinomial model more interpretable, we estimated the marginal willingness to trade treatments and different levels of attributes by changes in the 10-year OS. Most notably, if a treatment option causes moderate or severe long-term cognitive impairment, the 10-year OS rate should be improved by 10.4% (95% CI: 8.4 to 12.5%) and 25.1% (95% CI: 23.5 to 26.7%), respectively, to remain equally preferable to a treatment option causing mild cognitive impairment. For an 80–100% chance of premature menopause, the additional improvement in the 10-year OS rate was 5.8% (95% CI: 3.2 to 8.4%). For other negative outcomes and treatment choices (i.e. the constant), a small improvement in the 10-year OS rate (<5%) was found to be sufficient to justify the treatment option.

## Discussion

This study assessed healthcare professionals’ treatment preferences in view of the trade-offs between different outcome aspects for patients with HRD stage III HER2-negative breast cancer. Most healthcare professionals rated HDCT as experimental and had an average of 16 years of experience in treating stage III breast cancer patients. Furthermore, of the 24 medical oncologists, 23 (65.7%) had been educated on HDCT treatment, and 6 (25%) had performed HDCT as registered, ultimately responsible internists. Healthcare providers consider the 10-year OS and long-term cognitive impairment to be the most important attributes of treatment. Furthermore, we assessed individuals’ willingness to exchange a 10-year OS rate for other treatment attributes when other outcomes remained equal. The most noteworthy finding was that 25.1% (95% CI: 23.5 to 26.7%) additional improvement in the 10-year OS rate was required to justify accepting severe long-term cognitive impairment as a trade-off.

This is one of the first studies assessing the treatment (outcome) preferences of healthcare professionals regarding treatment choices (including HDCT) for patients with stage III breast cancer [33]. A previous unlabelled DCE with seven attributes investigated patients’ preferences in stage 3 and stage 4 breast cancer [34]. They found that patient preferences are heterogeneous but assigned great value to a longer ‘minimum life extension’, which ranged from 3 to 24 months. Other relatively important attributes to the choice of a treatment for these patients included out-of-pocket cost, route of administration, and the availability of reliable tests to help gauge treatment efficacy. Short-term treatment toxicity is relatively less important [34]. In contrast, breast cancer patients with metastasised (stage 4) hormone receptor-positive, HER-negative tumours seem to prioritise progression-free survival and the management of adverse events. In this study, however, the investigators did not use the grading system for adverse events to explore preferences, but explored specific adverse events [35]. It may therefore be possible that short-term toxicities may be more important in settings in which patients have a very short life expectancy or that healthcare providers score toxicities differently than patients.

The 10-year OS and exact effects of HDCT on cognition compared to those of standard of care are unknown and are currently under investigation in the SUBITO trial. Previously, Vollebergh et al. showed that 7-year recurrence-free survival improved from 30% with standard-dose chemotherapy to 78% with HDCT for patients with HRD tumours [1]. In this study, health-related quality of life (HRQoL) was worse after HDCT than after standard-dose chemotherapy; but after one year, the differences in HRQoL were negligible [36]. However, it became apparent in a follow-up study that two years after the last chemotherapy, cognitive impairment was present in 32% of the patients treated with high-dose chemotherapy, 17% of those treated with standard-dose chemotherapy, and 9% of the control patients [37]. Moreover, a study using multimodal magnetic resonance imaging (MRI) to study white and grey matter revealed that high-dose adjuvant chemotherapy may have substantial long-term detrimental effects on the brain compared to not receiving any chemotherapy [38]. Notably, the abovementioned studies investigated the effects of a more intensive HDCT regimen than the one currently under investigation in the SUBITO trial.

A recent review indicated that the adverse effects of chemotherapy on cognitive functioning are generally mild to moderate in severity and mainly concern learning and memory ability, executive function, and processing speed [23]. Various interventions are being developed and evaluated that target and manage cancer-related cognitive impairment [39, 40]. Cognitive rehabilitation programmes can help patients compensate for and cope with cognitive impairment by means of compensatory strategies [23]. Based on SUBITO study data, we hope to generate more information on whether and how HDCT affects different cognitive functions.

Due to limitations in our sample size, we were unable to conduct an analysis of the interaction between treatment attributes and healthcare providers’ characteristics, known as preference heterogeneity analysis [41]. However, this area of investigation holds potential for future studies, particularly when examining variations among different professions (e.g. medical oncologists, surgical oncologists, and oncology nurse practitioners), years of experience in the breast cancer field, whether the respondent had experience as the responsible internist, and the responses to the three questions related to ‘Rogers’ diffusion of innovation curve’ (majority (score ≤ 6), or innovator (score > 6)) [9]. For instance, it is plausible that oncologists and oncology nurse practitioners may have different perspectives on the short-term toxicity of chemotherapeutics compared to surgeons. Moreover, internists who administer HDCT may have a different view. Therefore, further investigation is warranted.

One unexpected result was a positive, although non-significant, sign of grade 3 toxicity. A possible explanation is that treatment descriptions, particularly details about duration and mention of HDCT, may create a correlation between toxicity and the alternative specific constant. To support this idea, we ran a different model including an interaction between the HDCT-specific constant and toxicity. In this new model, the signs of the coefficients of the toxicity levels for the standard of care were negative, as expected. Additionally, the signs of toxicity for HDCT were reversed, as before. None of these coefficients were significant.

In conclusion, insights into healthcare professionals’ treatment preferences and their assessment of various treatment outcomes serve as valuable resources for shared decision-making with patients [42]. Understanding healthcare providers’ preferences and priorities can provide guidance to professionals on shared decision-making, thus, encouraging collaborative and personalised decision-making processes aligned with individual patient values and preferences [42]. Particularly in complex scenarios, such as with stage III HER2-negative breast cancer patients with HRD tumours, using this understanding enriches discussions between patients and healthcare providers. Informed guidance ensures that treatment decisions, with their implications, are made in an informed manner, taking into account crucial treatment outcome possibilities.

## Limitations

Our study has some limitations. First, we enrolled 35 healthcare professionals in our study due to a moderate response rate (23.2%; 35 out of 151), particularly among surgical oncologists (16.7%; 8 out of 48), despite the development of a paper and online version of the DCE and several reminders. Second, in reality, additional factors are important for treatment choices in addition to our determined attributes and levels. Moreover, short-term treatment toxicities, cognitive impairment, and their corresponding levels could be described in more detail and tested in a future DCE to explore the relative importance of more specific outcomes. Third, although we have no reason to doubt the representativeness of the participants, we lack information on the non-respondents, limiting our ability to analyse participation patterns and generalise the findings beyond the subset of participants who participated in the study. However, the current study provided clear answers within the given framework and therefore provided valuable information on healthcare providers’ preferences. Last, there is some literature on patient preferences for stage III breast cancer, but not in the context of HDCT. It would be interesting to explore a similar DCE with treatment outcomes (and other modalities) within a patient population scheduled for HDCT.

## Conclusion

The effectiveness of the treatment (10-year OS rate) and the degree of long-term cognitive impairment were the most important attributes for healthcare providers to determine the most optimal treatment plan for patients with stage III breast cancer with HRD tumours. These two attributes combined affected more than 80% of the decision-making in this DCE. Furthermore, we found that there should be a considerable amount of absolute 10-year OS benefit to permit moderate and severe long-term cognitive impairment, that is, 10.4% and 25.1%, respectively, to remain equally preferable. Therefore, based on our sample, our conclusion is that healthcare professionals accept HDCT if the 10-year OS benefit sufficiently outweighs treatment-related long-term cognitive impairment. This information calls for further research into chemotherapy-related cognitive impairment and is helpful for guiding shared decision-making by highlighting the importance of understanding healthcare professionals’ preferences and priorities. This understanding can support more informed discussions about potential trade-offs among treatment options, including considerations of cognitive functioning.

## Supplementary Material

High-dose chemotherapy for patients with stage III breast cancer with homologous recombination deficiency: a discrete choice experiment among healthcare providers

## Data Availability

The Python code used for the analysis of this study can be obtained via reasonable request to the corresponding author.
