# Supplementary material for: High-dose chemotherapy for patients with stage III breast cancer with homologous recombination deficiency: a discrete choice experiment among healthcare providers
Source: Acta Oncol. 2024 Sep 10;63:40276. doi: 10.2340/1651-226X.2024.40276 (PMC11409819; doi:10.2340/1651-226X.2024.40276)
Supplement: High-dose chemotherapy for patients with stage III breast cancer with homologous recombination deficiency: a discrete choice experiment among healthcare providers [file AO-63-40276-s1.pdf]

Supplementary material has been published as submitted. It has not been copyedited, or typeset by Acta Oncologica

Appendix A. Materials used for the ranking exercise and the open discussion to determine attributes and levels (Dutch and English version).

Uit tabel 1 moeten we de meest relevante attributen kiezen, i.o.m DCE experts en artsen. Eventueel zouden er ook nog andere attributen bij kunnen komen. De attributen zijn verkregen uit de thema's van de content analyse en de literatuur. Ons doel is om met name de studievragen (direct) beantwoorden.

Studievragen:

''What are healthcare providers' preferences for the treatment of stage III breast cancer patients with a homologous recombination deficiency?''

''How do healthcare providers make trade-offs between different attributes when selecting a treatment for stage III breast cancer patients with a homologous recombination deficiency?"

Rangschikkingsoefening

- Wat zijn de belangrijkste overwegingen bij het kiezen van de meest geschikte behandeling voor deze patiëntengroep? Rank de attributen van 1 (meest belangrijk) tot 12 (minst belangrijk). Er kunnen additionele attributen worden toegevoegd aan de tabel.
- Wat zijn relevante 'levels' van deze attributen?
- Maak samen met de DCE expert/arts een of twee relevante keuzesets.

**Tabel 1. Attributen en alternatieven voor de behandeling van hoge risico borstkanker patiënten met een homologie recombinatie deficiëntie**	
**Attributen**	**Alternatieven/levels**
**Kans op 10-jaar overleving**	
Beide behandelingen	40% - 50% - 60% - 70% - 80%
**Kosten behandeling**	
Beide behandelingen	€25.000 - €35.000 - €45.000 - €55.000
**Lange termijn bijwerkingen**	
Kans op een secundaire maligniteit	

Beide behandelingen	5% - 15% - 20%
Kans op cognitieve problemen, i.e. concentratiestoornissen en/of geheugenproblemen	
Beide behandelingen	25% - 35% - 45%
Kans op onvruchtbaarheid door de behandeling	
Beide behandelingen	70% - 80% - 90% - 100%
Kans op een cardiovasculaire 'event'	
Beide behandelingen	5% – 10% – 15%
**Kans op een ernstig ongewenst ongeval tijdens de behandeling**	
Beide behandelingen	Zeer onwaarschijnlijk (~0%) – onwaarschijnlijk (0.5%-1%) – mogelijk (>1%)
**Kenmerken van de behandeling (fixed)**	
Hoge dosis chemotherapie	Duur van de behandeling is 16 weken; vier cycli (neo)adjuvante 'dose-dense' doxorubicine + cyclofosfamide waarvan de vierde met stamcelmobilisatie gevolgd door stamceloogsting, gevolgd door twee cycli geïntensiveerde alkylerende chemotherapie bestaande uit cyclofosfamide, thiotepa en carboplatine, afgesloten met stamcelteruggave.
Conventionele chemotherapie + Olaparib	Duur van de behandeling is 72 weken; vier cycli (neo)adjuvante 'dose-dense' doxorubicine + cyclofosfamide, gevolgd door vier cycli driewekelijks carboplatine gecombineerd met wekelijks paclitaxel, gevolgd door een jaar adjuvant olaparib.[1]
**Toxische effecten van de behandeling**	
Beide behandelingen	1. Gematigde kans op mucositis, diarree, extreme vermoeidheid, infectie, allergische reacties, en uitslag.

	2. Hoge kans op mucositis, diarree, extreme vermoeidheid, infectie, allergische reacties, en uitslag.<br>3. Zeer hoge kans op mucositis, diarree, extreme vermoeidheid, infectie, allergische reacties, en uitslag.
**Kwaliteit van leven na de behandeling**	
Beide behandelingen	1. Geen problemen met mobiliteit, verzorging, dagelijkste activiteiten, pijn of ongemak, angst of depressie.<br>2. Geen problemen met mobiliteit, verzorging, angst of depressie; Moeilijkheid met het uitvoeren van dagelijkse activiteiten en gematigde pijn of ongemak.<br>3. Geen problemen met mobiliteit, verzorging, angst of depressie; Ernstige moeilijkheden met het uitvoeren van dagelijkse activiteiten en gematigde pijn of ongemak.
**Organisatorische complexiteit (fixed)**	
Hoge dosis chemotherapie<br><br>Conventionele chemotherapie + Olaparib	Hoog, gecentraliseerd in beperkt aantal ziekenhuizen en een unieke samenwerking tussen hematologie en oncologie<br><br>Laag, standaardtherapie met jaar verlenging additionele medicatie
**Imago behandeling (fixed)**	

Hoge dosis chemotherapie<br><br><br><br>Conventionele chemotherapie + Olaparib	Negatief, door negatieve publicaties over geringe effectiviteit verleden, zware bijwerkingen en gefraudeerde studies<br><br>Positief, door positieve veelbelovende studies over aantoonbare effectiviteit en relatief geringe bijwerkingen
**Kans om terug te keren naar werk binnen een jaar na de behandeling**	
Beide behandelingen	Laag - Gemiddeld – Hoog

Translation

From table 1, we have to choose the most relevant attributes, in collaboration with DCE experts and healthcare providers. Other attributes could be added to the table if needed. The current attributes are obtained from the themes of the content analysis (i.e. qualitative paper) and a literature search. Our goal in the current study is to (directly) answer the study questions.

Study questions:

"What are healthcare providers' preferences for the treatment of stage III breast cancer patients with a homologous recombination deficiency?''

''How do healthcare providers make trade-offs between different attributes when selecting a treatment for stage III breast cancer patients with a homologous recombination deficiency?"

Ranking exercise

• What are the main considerations when choosing the best treatment for this patient group? Rank the attributes from 1 (most important) to 12 (least important). Add other additional attributes if necessary.

• What are relevant 'levels' of these attributes?

• Make one or two relevant choice sets together with the DCE expert/healthcare provider and see if any changes to the attribute (selection) are necessary.

**Table 1. Attributes and alternatives for the treatment of stage III breast cancer patients with a homologous recombination deficiency.**	
**Attributes**	**Alternative/levels**
**10-year overall survival rate**	
Both treatments	40% - 50% - 60% - 70% - 80%
**Cost of the treatment**	
Both treatments	€25.000 - €35.000 - €45.000 - €55.000
**Long-term side effects**	
Chance on a secondary malignity	

Both treatments	5% - 15% - 20%
Chance on cognitive impairment, i.e. concentration problems and/or memory problems	
Both treatments	25% - 35% - 45%
Chance on premature menopause due to the treatment	
Both treatments	70% - 80% - 90% - 100%
Chance on cardiovascular events	
Both treatments	5% – 10% – 15%
**Chance on a severe adverse event due to the treatment**	
Both treatments	Very unlikely (~0%) – unlikely (0.5%-1%) – possible (>1%)
**Characteristics of the treatment**	
High-dose chemotherapy	Duration of treatment is 16 weeks; four cycles of (neo)adjuvant 'dose-dense' doxorubicin + cyclophosphamide, the fourth with stem cell mobilization followed by stem cell harvesting, followed by two cycles of intensified alkylating chemotherapy consisting of cyclophosphamide, thiotepa and carboplatin, completed with stem cell restitution.
Standard-dose chemotherapy with olaparib	Duration of treatment is 72 weeks; four cycles of (neo)adjuvant dose-dense doxorubicin + cyclophosphamide, followed by four cycles of three-weekly carboplatin combined with weekly paclitaxel, followed by one year of adjuvant olaparib.
**Toxische effecten van de behandeling**	
Both treatments	1. Moderate risk of mucositis, diarrhea, extreme fatigue, infection, allergic reactions, and rash.

	2. High risk of mucositis, diarrhea, extreme fatigue, infection, allergic reactions, and rash.<br>3. Very high risk of mucositis, diarrhea, extreme fatigue, infection, allergic reactions, and rash.
**Quality of life after the treatment**	
Both treatments	1. No problems with mobility, grooming, daily activities, pain or discomfort, anxiety or depression.<br>2. No problems with mobility, grooming, anxiety or depression; Difficulty performing daily activities and moderate pain or discomfort.<br>3. No problems with mobility, grooming, anxiety or depression; Severe difficulty performing daily activities and moderate pain or discomfort.
**Organisational complexity (fixed)**	
High-dose chemotherapy	High, centralized in limited number of hospitals and a unique collaboration between hematology and oncology
Standard-dose chemotherapy with olaparib	Low, standard therapy with year extension of additional medication
**Perception of the treatment (fixed)**	
Hoge-dose chemotherapy	Negative, due to negative publications about low effectiveness in the past, serious side effects and fraudulent studies
Standard-dose chemotherapy with olaparib	Positive, due to positive promising studies on demonstrable effectiveness and relatively few side effects

**Chance to return-to-work 1 year after the treatment**	
Both treatments	Low – Medium - High

Appendix B. Questionnaire version A (Dutch and an English translation)

Beste deelnemer,

Welkom bij dit onderzoek naar de voorkeuren van behandelaars voor behandelingen bij stadium III borstkankerpatiënten met een homologe recombinatie deficiëntie (*BRCA1/2* kiembaanmutatie of BRCA-1 like). Dit onderzoek, dat deel uitmaakt van de SUBITO-studie, vergelijkt de behandelingen: 1) hoge dosis chemotherapie met autologe stamceltransplantatie (HDCT) en 2) standaard dosis chemotherapie verlengd met een jaar olaparib adjuvant. Het doel van deze vragenlijst is inzicht krijgen in welke mate bepaalde factoren de keuzes van behandelaars voor de bovenstaande patiëntengroep beïnvloeden in een aantal hypothetische scenario's. Dit kan helpen om tot een geïnformeerde gezamenlijke besluitvorming voor deze patiëntgroep te komen.

De vragenlijst bestaat uit drie delen met in totaal vijftien vragen en keuzesets. Kies bij elke vraag het antwoord dat **het meest op u van toepassing is**. Kruis hierbij één van de hokjes aan, tenzij anders aangegeven. Op eventueel aanwezige stippellijntjes kunt u uw eigen antwoord schrijven.

Er zijn geen 'goede' of 'foute' antwoorden: uw persoonlijke mening en voorkeur is belangrijk. **Wij verzoeken u geen vragen over te slaan**. Bij twijfel kiest u voor het antwoord dat het meest bij u past. Het invullen van deze vragenlijst kost ongeveer 15-20 minuten. Er zal vertrouwelijk met uw antwoorden worden omgegaan en uw antwoorden worden anoniem verwerkt, zodat alleen de onderzoekers Valesca Retèl, Joost Verbeek en Hanna Wijnja kunnen herleiden van wie de antwoorden afkomstig zijn.

Wij danken u bij voorbaat hartelijk voor het invullen van deze vragenlijst!

Prof. Dr. Sabine Linn
Hoofdonderzoeker SUBITO-studie

Dr. Valesca Retèl
Senior onderzoeker Health Technology Assessment
NKI-AVL

Drs. Joost Verbeek
PhD-kandidaat Health Technology Assessment
NKI-AVL

Voor eventuele vragen en opmerkingen kunt u contact opnemen met Joost Verbeek (j.verbeek@nki.nl)

DEEL 1: PERSOONSGEGEVENS

1. Wat is uw geslacht?

☐ Man

☐ Vrouw

☐ Anders, namelijk...

2. Wat is uw leeftijd?.......................................

3. Wat is uw functie binnen de mammazorg?

☐ Medisch oncoloog

☐ Chirurgisch oncoloog

☐ Hematoloog

☐ Verpleegkundig specialist

☐ Anders, namelijk...

4. Hoe lang bent u actief binnen de mammazorg?................................

5. Voor welk type instelling bent u werkzaam?

☐ Academisch ziekenhuis

☐ STZ-ziekenhuis

☐ Perifeer ziekenhuis met opleiding interne geneeskunde bevoegdheid

☐ Perifeer ziekenhuis zonder opleiding interne geneeskunde bevoegdheid

☐ Comprehensive Cancer Center

☐ Anders, namelijk...

6. Heeft u ervaring met het uitvoeren van autologe stamceltransplantaties?

☐ Ja

☐ Nee

7. Hoeveel stadium III borstkankerpatiënten behandelt u ongeveer per jaar als individu?

.... borstkankerpatiënten

DEEL 2: BEHANDELVOORKEUREN

Op pagina's 5 tot en met 12 wordt u telkens een keuzeset voorgelegd. Deze keuzeset heeft twee opties, optie A en optie B, met daaronder weergegeven hoe zij scoren op bepaalde aspecten die belangrijk zijn voor de keuze van de behandeling (zogenaamde attributen) van stadium III borstkanker met homologe recombinatie deficiëntie. **Een uitleg van de attributen in de keuzesets:**

**Kenmerken van de behandeling**	
Hoge dosis chemotherapie	Duur van de behandeling is 16 weken; vier cycli (neo)adjuvante 'dose-dense' doxorubicine + cyclofosfamide waarvan de vierde met stamcelmobilisatie gevolgd door stamceloogsting, gevolgd door twee cycli geïntensiveerde alkylerende chemotherapie bestaande uit cyclofosfamide, thiotepa en carboplatine, afgesloten met stamcelteruggave.[1]
Standaard dosis chemotherapie met olaparib	Duur van de behandeling is 72 weken; vier cycli (neo)adjuvante 'dose-dense' doxorubicine + cyclofosfamide, gevolgd door vier cycli driewekelijks carboplatine gecombineerd met wekelijks paclitaxel, gevolgd door een jaar adjuvant olaparib.[1]
**10-jaar overlevingskans na diagnose[1,2]**	
50% - 60% - 70% - 80% - 90%	Kans op overleving 10 jaar na diagnose.
**Bijwerkingen tijdens de behandeling[3]**	
Graad 2	Behandeling veroorzaakt enkel milde tot matige bijwerkingen, geen tot minimale interventie nodig, geen tot minimale limitatie dagelijkse activiteiten.
Graad 3	Behandeling veroorzaakt ernstige bijwerkingen, (verlenging van) ziekenhuisopname nodig, behoorlijke limitatie dagelijkse activiteiten.
Graad 4	Behandeling veroorzaakt levensbedreigende bijwerkingen, urgente interventie nodig, ernstige limitatie zelfstandigheid dagelijkse activiteiten.
**Cognitieve bijwerkingen op de lange termijn**	
Mild	De behandeling veroorzaakt geen tot milde cognitieve problemen op de lange termijn.
Matig	De behandeling veroorzaakt gematigde cognitieve problemen op de lange termijn.
Ernstig	De behandeling veroorzaakt ernstige cognitieve problemen op de lange termijn.
**Kans om postmenopauzaal te worden door de behandeling[4]**	
10-30%	Kans dat een patiënt postmenopauzaal wordt na de behandeling.
45-65%	Kans dat een patiënt postmenopauzaal wordt na de behandeling.
80-100%	Kans dat een patiënt postmenopauzaal wordt na de behandeling.

*1-4: Zie bladzijde 16 voor referentielijst

VOORBEELD

Voorbeeld van een keuzeset

De vraag bij elke keuzeset: ''Een 40-jarige patiënte is gediagnosticeerd met stadium III borstkanker met homologe recombinatie deficiëntie. Twee behandelingen die verschillende uitkomsten opleveren zijn mogelijk. Welke optie zou u kiezen voor deze patiënt op basis van onderstaande gegevens?''

**Attribuut**	**Optie A: Hoge dosis chemotherapie (16 weken)**	**Optie B: Standaard dosis chemotherapie met olaparib (72 weken)**
**10-jaar overlevingskans na diagnose**	50%	50%
**Bijwerkingen tijdens de behandeling**	Graad 3	Graad 4
**Bijwerkingen op cognitie op de lange termijn**	Ernstig	Matig
**Kans om postmenopauzaal te worden door de behandeling**	10-30%	10-30%
**Keuze**	☐ **Optie A**	☐ **Optie B**

Op de komende pagina's leggen wij 15 scenario's aan u voor. Wij vragen u de gepresenteerde informatie in de scenario's goed door te nemen en op basis van deze informatie telkens één keuze te maken voor ofwel optie A ofwel optie B. Hierbij kunt u gebruik maken van de **uitleg van de attributen op pagina 3.** Het is belangrijk dat u geen scenario overslaat. Er zijn geen 'foute' antwoorden, het gaat om uw persoonlijke voorkeur.

De verwachte uitkomsten van de behandelingen in de keuzesets zijn hypothetisch en zijn dus mogelijk niet in overeenstemming met de realiteit.

Keuzeset 1

Een 40-jarige patiënte is gediagnosticeerd met stadium III borstkanker met homologe recombinatie deficiëntie. Twee behandelingen die verschillende uitkomsten opleveren zijn mogelijk. Welke optie zou u kiezen voor deze patiënt op basis van onderstaande gegevens?

Attribuut	Optie A: Hoge dosis chemotherapie (16 weken)	Optie B: Standaard dosis chemotherapie met olaparib (72 weken)
**10-jaar overlevingskans na diagnose**	50%	90%
**Bijwerkingen tijdens de behandeling**	Graad 3	Graad 3
**Bijwerkingen op cognitie op de lange termijn**	Mild	Ernstig
**Kans om postmenopauzaal te worden door de behandeling**	10-30%	10-30%
**Keuze**	☐ **Optie A**	☐ **Optie B**

Keuzeset 2

Een 40-jarige patiënte is gediagnosticeerd met stadium III borstkanker met homologe recombinatie deficiëntie. Twee behandelingen die verschillende uitkomsten opleveren zijn mogelijk. Welke optie zou u kiezen voor deze patiënt op basis van onderstaande gegevens?

**Attribuut**	**Optie A: Hoge dosis chemotherapie (16 weken)**	**Optie B: Standaard dosis chemotherapie met olaparib (72 weken)**
**10-jaar overlevingskans na diagnose**	60%	70%
**Bijwerkingen tijdens de behandeling**	Graad 2	Graad 4
**Bijwerkingen op cognitie op de lange termijn**	Ernstig	Mild
**Kans om postmenopauzaal te worden door de behandeling**	10-30%	10-30%
**Keuze**	☐ **Optie A**	☐ **Optie B**

Keuzeset 3

Een 40-jarige patiënte is gediagnosticeerd met stadium III borstkanker met homologe recombinatie deficiëntie. Twee behandelingen die verschillende uitkomsten opleveren zijn mogelijk. Welke optie zou u kiezen voor deze patiënt op basis van onderstaande gegevens?

**Attribuut**	**Optie A: Hoge dosis chemotherapie (16 weken)**	**Optie B: Standaard dosis chemotherapie met olaparib (72 weken)**
**10-jaar overlevingskans na diagnose**	90%	80%
**Bijwerkingen tijdens de behandeling**	Graad 4	Graad 2
**Bijwerkingen op cognitie op de lange termijn**	Ernstig	Mild
**Kans om postmenopauzaal te worden door de behandeling**	10-30%	10-30%
**Keuze**	☐ **Optie A**	☐ **Optie B**

Keuzeset 4

Een 40-jarige patiënte is gediagnosticeerd met stadium III borstkanker met homologe recombinatie deficiëntie. Twee behandelingen die verschillende uitkomsten opleveren zijn mogelijk. Welke optie zou u kiezen voor deze patiënt op basis van onderstaande gegevens?

**Attribuut**	**Optie A: Hoge dosis chemotherapie (16 weken)**	**Optie B: Standaard dosis chemotherapie met olaparib (72 weken)**
**10-jaar overlevingskans na diagnose**	70%	80%
**Bijwerkingen tijdens de behandeling**	Graad 2	Graad 2
**Bijwerkingen op cognitie op de lange termijn**	Mild	Ernstig
**Kans om postmenopauzaal te worden door de behandeling**	45-65%	10-30%
**Keuze**	☐ **Optie A**	☐ **Optie B**

Keuzeset 5

Een 40-jarige patiënte is gediagnosticeerd met stadium III borstkanker met homologe recombinatie deficiëntie. Twee behandelingen die verschillende uitkomsten opleveren zijn mogelijk. Welke optie zou u kiezen voor deze patiënt op basis van onderstaande gegevens?

**Attribuut**	**Optie A: Hoge dosis chemotherapie (16 weken)**	**Optie B: Standaard dosis chemotherapie met olaparib (72 weken)**
**10-jaar overlevingskans na diagnose**	70%	90%
**Bijwerkingen tijdens de behandeling**	Graad 2	Graad 4
**Bijwerkingen op cognitie op de lange termijn**	Mild	Mild
**Kans om postmenopauzaal te worden door de behandeling**	45-65%	45-65%
**Keuze**	☐ **Optie A**	☐ **Optie B**

Keuzeset 6

Een 40-jarige patiënte is gediagnosticeerd met stadium III borstkanker met homologe recombinatie deficiëntie. Twee behandelingen die verschillende uitkomsten opleveren zijn mogelijk. Welke optie zou u kiezen voor deze patiënt op basis van onderstaande gegevens?

**Attribuut**	**Optie A: Hoge dosis chemotherapie (16 weken)**	**Optie B: Standaard dosis chemotherapie met olaparib (72 weken)**
**10-jaar overlevingskans na diagnose**	80%	50%
**Bijwerkingen tijdens de behandeling**	Graad 2	Graad 2
**Bijwerkingen op cognitie op de lange termijn**	Matig	Ernstig
**Kans om postmenopauzaal te worden door de behandeling**	45-65%	10-30%
**Keuze**	☐ **Optie A**	☐ **Optie B**

Keuzeset 7

Een 40-jarige patiënte is gediagnosticeerd met stadium III borstkanker met homologe recombinatie deficiëntie. Twee behandelingen die verschillende uitkomsten opleveren zijn mogelijk. Welke optie zou u kiezen voor deze patiënt op basis van onderstaande gegevens?

**Attribuut**	**Optie A: Hoge dosis chemotherapie (16 weken)**	**Optie B: Standaard dosis chemotherapie met olaparib (72 weken)**
**10-jaar overlevingskans na diagnose**	90%	70%
**Bijwerkingen tijdens de behandeling**	Graad 4	Graad 3
**Bijwerkingen op cognitie op de lange termijn**	Matig	Ernstig
**Kans om postmenopauzaal te worden door de behandeling**	45-65%	45-65%
**Keuze**	☐ **Optie A**	☐ **Optie B**

Keuzeset 8

Een 40-jarige patiënte is gediagnosticeerd met stadium III borstkanker met homologe recombinatie deficiëntie. Twee behandelingen die verschillende uitkomsten opleveren zijn mogelijk. Welke optie zou u kiezen voor deze patiënt op basis van onderstaande gegevens?

**Attribuut**	**Optie A: Hoge dosis chemotherapie (16 weken)**	**Optie B: Standaard dosis chemotherapie met olaparib (72 weken)**
**10-jaar overlevingskans na diagnose**	60%	90%
**Bijwerkingen tijdens de behandeling**	Graad 3	Graad 4
**Bijwerkingen op cognitie op de lange termijn**	Ernstig	Matig
**Kans om postmenopauzaal te worden door de behandeling**	45-65%	10-30%
**Keuze**	☐ **Optie A**	☐ **Optie B**

Keuzeset 9

Een 40-jarige patiënte is gediagnosticeerd met stadium III borstkanker met homologe recombinatie deficiëntie. Twee behandelingen die verschillende uitkomsten opleveren zijn mogelijk. Welke optie zou u kiezen voor deze patiënt op basis van onderstaande gegevens?

**Attribuut**	**Optie A: Hoge dosis chemotherapie (16 weken)**	**Optie B: Standaard dosis chemotherapie met olaparib (72 weken)**
**10-jaar overlevingskans na diagnose**	80%	60%
**Bijwerkingen tijdens de behandeling**	Graad 4	Graad 3
**Bijwerkingen op cognitie op de lange termijn**	Ernstig	Matig
**Kans om postmenopauzaal te worden door de behandeling**	45-65%	10-30%
**Keuze**	☐ **Optie A**	☐ **Optie B**

Keuzeset 10

Een 40-jarige patiënte is gediagnosticeerd met stadium III borstkanker met homologe recombinatie deficiëntie. Twee behandelingen die verschillende uitkomsten opleveren zijn mogelijk. Welke optie zou u kiezen voor deze patiënt op basis van onderstaande gegevens?

**Attribuut**	**Optie A: Hoge dosis chemotherapie (16 weken)**	**Optie B: Standaard dosis chemotherapie met olaparib (72 weken)**
**10-jaar overlevingskans na diagnose**	60%	50%
**Bijwerkingen tijdens de behandeling**	Graad 4	Graad 3
**Bijwerkingen op cognitie op de lange termijn**	Mild	Mild
**Kans om postmenopauzaal te worden door de behandeling**	80-100%	10-30%
**Keuze**	☐ **Optie A**	☐ **Optie B**

Keuzeset 11

Een 40-jarige patiënte is gediagnosticeerd met stadium III borstkanker met homologe recombinatie deficiëntie. Twee behandelingen die verschillende uitkomsten opleveren zijn mogelijk. Welke optie zou u kiezen voor deze patiënt op basis van onderstaande gegevens?

**Attribuut**	**Optie A: Hoge dosis chemotherapie (16 weken)**	**Optie B: Standaard dosis chemotherapie met olaparib (72 weken)**
**10-jaar overlevingskans na diagnose**	90%	70%
**Bijwerkingen tijdens de behandeling**	Graad 4	Graad 2
**Bijwerkingen op cognitie op de lange termijn**	Mild	Matig
**Kans om postmenopauzaal te worden door de behandeling**	80-100%	80-100%
**Keuze**	☐ **Optie A**	☐ **Optie B**

Keuzeset 12

Een 40-jarige patiënte is gediagnosticeerd met stadium III borstkanker met homologe recombinatie deficiëntie. Twee behandelingen die verschillende uitkomsten opleveren zijn mogelijk. Welke optie zou u kiezen voor deze patiënt op basis van onderstaande gegevens?

**Attribuut**	**Optie A: Hoge dosis chemotherapie (16 weken)**	**Optie B: Standaard dosis chemotherapie met olaparib (72 weken)**
**10-jaar overlevingskans na diagnose**	50%	50%
**Bijwerkingen tijdens de behandeling**	Graad 2	Graad 2
**Bijwerkingen op cognitie op de lange termijn**	Matig	Ernstig
**Kans om postmenopauzaal te worden door de behandeling**	80-100%	45-65%
**Keuze**	☐ **Optie A**	☐ **Optie B**

Keuzeset 13

Een 40-jarige patiënte is gediagnosticeerd met stadium III borstkanker met homologe recombinatie deficiëntie. Twee behandelingen die verschillende uitkomsten opleveren zijn mogelijk. Welke optie zou u kiezen voor deze patiënt op basis van onderstaande gegevens?

**Attribuut**	**Optie A: Hoge dosis chemotherapie (16 weken)**	**Optie B: Standaard dosis chemotherapie met olaparib (72 weken)**
**10-jaar overlevingskans na diagnose**	70%	70%
**Bijwerkingen tijdens de behandeling**	Graad 2	Graad 3
**Bijwerkingen op cognitie op de lange termijn**	Matig	Matig
**Kans om postmenopauzaal te worden door de behandeling**	80-100%	10-30%
**Keuze**	☐ **Optie A**	☐ **Optie B**

Keuzeset 14

Een 40-jarige patiënte is gediagnosticeerd met stadium III borstkanker met homologe recombinatie deficiëntie. Twee behandelingen die verschillende uitkomsten opleveren zijn mogelijk. Welke optie zou u kiezen voor deze patiënt op basis van onderstaande gegevens?

**Attribuut**	**Optie A: Hoge dosis chemotherapie (16 weken)**	**Optie B: Standaard dosis chemotherapie met olaparib (72 weken)**
**10-jaar overlevingskans na diagnose**	50%	60%
**Bijwerkingen tijdens de behandeling**	Graad 2	Graad 4
**Bijwerkingen op cognitie op de lange termijn**	Ernstig	Mild
**Kans om postmenopauzaal te worden door de behandeling**	80-100%	80-100%
**Keuze**	☐ **Optie A**	☐ **Optie B**

Keuzeset 15

Een 40-jarige patiënte is gediagnosticeerd met stadium III borstkanker met homologe recombinatie deficiëntie. Twee behandelingen die verschillende uitkomsten opleveren zijn mogelijk. Welke optie zou u kiezen voor deze patiënt op basis van onderstaande gegevens?

**Attribuut**	**Optie A: Hoge dosis chemotherapie (16 weken)**	**Optie B: Standaard dosis chemotherapie met olaparib (72 weken)**
**10-jaar overlevingskans na diagnose**	70%	50%
**Bijwerkingen tijdens de behandeling**	Graad 4	Graad 3
**Bijwerkingen op cognitie op de lange termijn**	Ernstig	Ernstig
**Kans om postmenopauzaal te worden door de behandeling**	80-100%	80-100%
**Keuze**	☐ **Optie A**	☐ **Optie B**

DEEL 3: OVERIGE VRAGEN

8. Bij het kiezen van de meest belangrijke attributen hebben we voor deze vragenlijst de overlevingskans, bijwerkingen tijdens de behandeling, en bijwerkingen op cognitie en de menopauze gebruikt voor de keuzesets. Mist u nog andere belangrijke attributen die u graag in de keuzesets had willen zien?...

9. Hoe zou u de status van hoge dosis chemotherapie met autologe stamceltransplantatie (HDCT) voor stadium III borstkanker patiënten met een homologe recombinatie deficiëntie (momenteel) beoordelen?

   ☐ Experimenteel

   ☐ Onbewezen

   ☐ Bewezen

   ☐ Anders, namelijk...

10. Waar baseert u die beoordeling over HDCT op (momenteel)? Meerdere antwoorden mogelijk

    ☐ Literatuur

    ☐ Beroepsgroep

    ☐ Prominente figuren

    ☐ Ervaring

    ☐ Anders, namelijk..

11. Bent u in aanraking gekomen met HDCT tijdens uw opleiding?

    ☐ Ja

    ☐ Nee

12. Heeft u als geregistreerd, eindverantwoordelijk internist/chirurg HDCT uitgevoerd?

    ☐ Ja

    ☐ Nee

    ☐ Niet van toepassing

Tot slot een aantal stellingen over het toepassen van **nieuwe behandelingen in het algemeen** (klik op de cirkel van uw keuze en druk op ‘x’ op uw toetsenbord):

13. Ik dien een behandeling toe meteen als er bewijs is voor haar effectiviteit, ook als collega’s (in andere centra) hieraan twijfelen

Helemaal mee oneens ○ ○ ○ ○ ○ Helemaal mee eens

14. Ik dien een nieuwe behandeling pas toe wanneer collega’s (in andere centra) hier goede ervaringen mee hebben

Helemaal mee oneens ○ ○ ○ ○ ○ Helemaal mee eens

15. Ik vertrouw op de mening over nieuwe behandelingen van prominente figuren in mijn vakgebied

Helemaal mee oneens ○ ○ ○ ○ ○ Helemaal mee eens

AFSLUITING

Mogen wij u benaderen bij eventuele onduidelijkheden over de ingevulde vragenlijst?

☐ Nee

☐ Ja, ik ben bereikbaar op telefoonnummer of e-mail: …………………………………………………

Wilt u een samenvatting ontvangen van de resultaten ontvangen?

☐ Ja, ik ontvang graag een samenvatting

☐ Nee, ik hoef geen samenvatting te ontvangen

U kunt hier eventuele opmerkingen noteren over de vragenlijst:

………

………

………

………

Wij willen u nogmaals hartelijk bedanken voor uw medewerking!

Referentielijst

1. Vliek, S. B., Jager, A., Jongen-Lavrencic, M., Kroep, J. R., Gort, E. H., Tjan-Heijen, V. C. G., Konings, I. R., Kuip, E. J. M., Wymenga, A. N. M., Schröder, C. P., & Linn, S. C. (2018). SUBITO trial: can we improve the cure rate of stage III breast cancer with BRCA function loss substantially using intensified alkylating chemotherapy with autologous stem cell transplantation? *Nederlands Tijdschrift Voor Oncologie, 15,* 316–320.

2. Robson, M. E., Tung, N., Conte, P., Im, S. A., Senkus, E., Xu, B., ... & Wu, W. (2019). OlympiAD final overall survival and tolerability results: Olaparib versus chemotherapy treatment of physician's choice in patients with a germline BRCA mutation and HER2-negative metastatic breast cancer. *Annals of Oncology, 30*(4), 558-566.

3. National Cancer Institute,National Institutes of Health, US Department of Health and Human Services. Common Terminology Criteria for Adverse Events (CTCAE), Version 4.0. NIH publication 09-7473. Published May 29, 2009; Revised June 14, 2010. http://evs.nci.nih.gov/ftp1/CTCAE/CTCAE _4.03_2010-06-14_QuickReference_5x7.pdf. Accessed May 12, 202.

4. Blumenfeld, Z. (2012). Chemotherapy and fertility. *Best Practice & Research Clinical Obstetrics & Gynaecology*, *26*(3), 379-390

Dear participant,

Welcome to this study on treatment preferences for stage III breast cancer patients with a homologous recombination deficiency (*BRCA1/2* germline mutation or BRCA1-like). This study, which is part of the SUBITO trial, compares two treatments: 1) high-dose chemotherapy with autologous stem cell transplantation (HDCT) and 2) standard-dose chemotherapy extended with one year of adjuvant olaparib. The aim of this questionnaire is to gain insight into the treatment preferences of healthcare professionals for this patient population in a number of hypothetical scenarios. This may help with informed shared decision-making for this patient group.

The questionnaire consists of three parts with a total of fifteen questions and choices. For each question, **choose the answer that most applies to you**. Tick only one of the boxes, unless otherwise indicated. You can write answers of the open questions on the dotted lines.

There are no 'right' or 'wrong' answers: your personal opinion and preference is important. **We ask that you do not skip any questions**. When in doubt, choose the answer that suits you best. Completing this questionnaire will take approximately 15-20 minutes. Your answers are confidential and will be processed anonymously.

Thank you in advance for completing this questionnaire!

Prof. dr. dr. Sabine Linn
Principal investigator of SUBITO study

dr. Valesca Retèl
Senior researcher Health Technology Assessment
NKI-AVL

drs. Joost Verbeek
PhD candidate Health Technology Assessment
NKI-AVL

For any questions, please contact Joost Verbeek (j.verbeek@nki.nl)

PART 1: DEMOGRAPHICS

1. What is your sex?

☐ Man

☐ Woman

☐ Other, namely…………………………………………

2. Wat is your age?………………………………….

3. What is your occupation within breast care?

☐ Medical oncologist

☐ Surgical oncologist

☐ Hematologist

☐ Specialized breast care nurse

☐ Other, namely…………………………………………

4. How long have you been active within breast care?……………………………

5. What type of institution do you work for?

☐ Academic hospital

☐ Tertiary Medical Teaching hospital (‘STZ-hospital’)

☐ Peripheral hospital with training in internal medicine

☐ Peripheral hospital without training in internal medicine

☐ Comprehensive Cancer Center

☐ Other, namely………………………………………..

6. Do you have experience performing autologous stem cell transplants?

☐ Yes

☐ No

7. Approximately how many stage III breast cancer patients do you treat per year as an individual? ……………. breast cancer patients

DEEL 2: BEHANDELVOORKEUREN

You will be presented with different choice sets on pages 5 to 12. Each choice set has two options, option A and option B, showing below every choice set how they score on certain aspects that are important for the choice of the treatment (so-called attributes) of stage III breast cancer with a homologous recombination deficiency. **An explanation of the attributes in the choice sets:**

**Features of the treatment**	
High-dose chemotherapy	Duration of treatment is 16 weeks; four cycles of (neo)adjuvant ‘dose-dense’ doxorubicin + cyclophosphamide, the fourth with stem cell mobilization followed by stem cell harvesting, followed by two cycles of intensified alkylating chemotherapy consisting of cyclophosphamide, thiotepa and carboplatin, completed with stem cell restitution.[1]
Standard-dose chemotherapy with olaparib	Duration of treatment is 72 weeks; four cycles of (neo)adjuvant dose-dense doxorubicin + cyclophosphamide, followed by four cycles of three-weekly carboplatin combined with weekly paclitaxel, followed by one year of adjuvant olaparib.[1]
**10-year overall survival after diagnosis**[1,2]	
50% - 60% - 70% - 80% - 90%	Chance of survival 10 years after diagnosis
**Side-effects during the treatment**[3]	
Grade 2	Treatment causes only mild to moderate side effects, no to minimal intervention required, no to minimal limitation of daily activities.
Grade 3	Treatment causes serious side effects, requires (extension of) hospitalization, considerable limitation of daily activities.
Grade 4	Treatment causes life-threatening side effects, urgent intervention is needed, severe limitation of daily activities.
**Long-term cognitive side-effects**	
Mild	The treatment causes no to mild long-term cognitive problems.
Moderate	The treatment causes moderate long-term cognitive problems.
Severe	The treatment causes serious long-term cognitive problems.
**Chance of premature menopause**[4]	
10-30%	Chance of a patient becoming postmenopausal after treatment.
45-65%	Chance of a patient becoming postmenopausal after treatment.
80-100%	Chance of a patient becoming postmenopausal after treatment.

EXAMPLE

Example of a choice set:

The question for each choice set: "A 40-year-old patient has been diagnosed with stage III breast cancer with homologous recombination deficiency. Two treatments with different outcomes are possible. Which option would you choose for this patient based on the information below?"

**Attributes**	**Option A: High-dose chemotherapy with stem cell rescue (16 weeks)**	**Option B: Standard-dose chemotherapy plus olaparib (72 weeks)**
**10-year survival rate after diagnosis**	70%	50%
**Side-effects during treatment**	Grade 3	Grade 4
**Long-term side-effects on cognition**	Severe	Moderate
**Chance of premature menopause**	10-30%	10-30%
**Choice**	☐ **Option A**	☐ **Option B**

On the following pages we present 15 scenarios to you. We ask that you to carefully read the information presented in the scenarios and, based on this information, make a choice between either option A or option B. You can use the explanation of the attributes on page 3. It is important that you don't skip a scenario. There are no 'wrong' answers, we are interested in your personal preference.

The expected outcomes of the treatments in the choice sets are hypothetical and thus may not correspond to reality.

Keuzeset 1

The question for each choice set: "A 40-year-old patient has been diagnosed with stage III breast cancer with homologous recombination deficiency. Two treatments with different outcomes are possible. Which option would you choose for this patient based on the information below?"

**Attributes**	**Option A: High-dose chemotherapy with stem cell rescue (16 weeks)**	**Optie B: Standard-dose chemotherapy plus olaparib (72 weeks)**
**10-year survival rate after diagnosis**	50%	90%
**Side-effects during treatment**	Grade 3	Grade 3
**Long-term side-effects on cognition**	Mild	Severe
**Chance of premature menopause**	10-30%	10-30%
**Choice**	☐ **Option A**	☐ **Option B**

Keuzeset 2

The question for each choice set: “A 40-year-old patient has been diagnosed with stage III breast cancer with homologous recombination deficiency. Two treatments with different outcomes are possible. Which option would you choose for this patient based on the information below?”

**Attributes**	**Option A: High-dose chemotherapy with stem cell rescue (16 weeks)**	**Optie B: Standard-dose chemotherapy plus olaparib (72 weeks)**
**10-year survival rate after diagnosis**	60%	70%
**Side-effects during treatment**	Grade 2	Grade 4
**Long-term side-effects on cognition**	Severe	Mild
**Chance of premature menopause**	10-30%	10-30%
**Choice**	☐ **Option A**	☐ **Option B**

Keuzeset 3

The question for each choice set: “A 40-year-old patient has been diagnosed with stage III breast cancer with homologous recombination deficiency. Two treatments with different outcomes are possible. Which option would you choose for this patient based on the information below?”

**Attributes**	**Option A: High-dose chemotherapy with stem cell rescue (16 weeks)**	**Optie B: Standard-dose chemotherapy plus olaparib (72 weeks)**
**10-year survival rate after diagnosis**	90%	80%
**Side-effects during treatment**	Grade 4	Grade 2
**Long-term side-effects on cognition**	Severe	Mild
**Chance of premature menopause**	10-30%	10-30%
**Choice**	☐ **Option A**	☐ **Option B**

Keuzeset 4

The question for each choice set: "A 40-year-old patient has been diagnosed with stage III breast cancer with homologous recombination deficiency. Two treatments with different outcomes are possible. Which option would you choose for this patient based on the information below?"

**Attributes**	**Option A: High-dose chemotherapy with stem cell rescue (16 weeks)**	**Optie B: Standard-dose chemotherapy plus olaparib (72 weeks)**
**10-year survival rate after diagnosis**	70%	80%
**Side-effects during treatment**	Grade 2	Grade 2
**Long-term side-effects on cognition**	Mild	Severe
**Chance of premature menopause**	45-65%	10-30%
**Choice**	☐ **Option A**	☐ **Option B**

Keuzeset 5

The question for each choice set: "A 40-year-old patient has been diagnosed with stage III breast cancer with homologous recombination deficiency. Two treatments with different outcomes are possible. Which option would you choose for this patient based on the information below?"

**Attributes**	**Option A: High-dose chemotherapy with stem cell rescue (16 weeks)**	**Optie B: Standard-dose chemotherapy plus olaparib (72 weeks)**
**10-year survival rate after diagnosis**	70%	90%
**Side-effects during treatment**	Grade 2	Grade 4
**Long-term side-effects on cognition**	Mild	Mild
**Chance of premature menopause**	45-65%	45-65%
**Choice**	☐ **Option A**	☐ **Option B**

Keuzeset 6

The question for each choice set: "A 40-year-old patient has been diagnosed with stage III breast cancer with homologous recombination deficiency. Two treatments with different outcomes are possible. Which option would you choose for this patient based on the information below?"

Attributes	Option A: High-dose chemotherapy with stem cell rescue (16 weeks)	Optie B: Standard-dose chemotherapy plus olaparib (72 weeks)
**10-year survival rate after diagnosis**	80%	50%
**Side-effects during treatment**	Grade 2	Grade 2
**Long-term side-effects on cognition**	Moderate	Severe
**Chance of premature menopause**	45-65%	10-30%
**Choice**	☐ **Option A**	☐ **Option B**

Keuzeset 7

The question for each choice set: "A 40-year-old patient has been diagnosed with stage III breast cancer with homologous recombination deficiency. Two treatments with different outcomes are possible. Which option would you choose for this patient based on the information below?"

Attributes	Option A: High-dose chemotherapy with stem cell rescue (16 weeks)	Optie B: Standard-dose chemotherapy plus olaparib (72 weeks)
**10-year survival rate after diagnosis**	90%	70%
**Side-effects during treatment**	Grade 4	Grade 3
**Long-term side-effects on cognition**	Moderate	Severe
**Chance of premature menopause**	45-65%	45-65%
**Choice**	☐ **Option A**	☐ **Option B**

Keuzeset 8

The question for each choice set: “A 40-year-old patient has been diagnosed with stage III breast cancer with homologous recombination deficiency. Two treatments with different outcomes are possible. Which option would you choose for this patient based on the information below?”

**Attributes**	**Option A: High-dose chemotherapy with stem cell rescue (16 weeks)**	**Optie B: Standard-dose chemotherapy plus olaparib (72 weeks)**
**10-year survival rate after diagnosis**	60%	90%
**Side-effects during treatment**	Grade 3	Grade 4
**Long-term side-effects on cognition**	Severe	Moderate
**Chance of premature menopause**	45-65%	10-30%
**Choice**	☐ **Option A**	☐ **Option B**

Keuzeset 9

The question for each choice set: “A 40-year-old patient has been diagnosed with stage III breast cancer with homologous recombination deficiency. Two treatments with different outcomes are possible. Which option would you choose for this patient based on the information below?”

**Attributes**	**Option A: High-dose chemotherapy with stem cell rescue (16 weeks)**	**Optie B: Standard-dose chemotherapy plus olaparib (72 weeks)**
**10-year survival rate after diagnosis**	80%	60%
**Side-effects during treatment**	Grade 4	Grade 3
**Long-term side-effects on cognition**	Severe	Moderate
**Chance of premature menopause**	45-65%	10-30%
**Choice**	☐ **Option A**	☐ **Option B**

Keuzeset 10

The question for each choice set: “A 40-year-old patient has been diagnosed with stage III breast cancer with homologous recombination deficiency. Two treatments with different outcomes are possible. Which option would you choose for this patient based on the information below?”

**Attributes**	**Option A: High-dose chemotherapy with stem cell rescue (16 weeks)**	**Optie B: Standard-dose chemotherapy plus olaparib (72 weeks)**
**10-year survival rate after diagnosis**	60%	50%
**Side-effects during treatment**	Grade 4	Grade 3
**Long-term side-effects on cognition**	Mild	Mild
**Chance of premature menopause**	80-100%	10-30%
**Choice**	☐ **Option A**	☐ **Option B**

Keuzeset 11

The question for each choice set: “A 40-year-old patient has been diagnosed with stage III breast cancer with homologous recombination deficiency. Two treatments with different outcomes are possible. Which option would you choose for this patient based on the information below?”

**Attributes**	**Option A: High-dose chemotherapy with stem cell rescue (16 weeks)**	**Optie B: Standard-dose chemotherapy plus olaparib (72 weeks)**
**10-year survival rate after diagnosis**	90%	70%
**Side-effects during treatment**	Grade 4	Grade 2
**Long-term side-effects on cognition**	Mild	Moderate
**Chance of premature menopause**	80-100%	80-100%
**Choice**	☐ **Option A**	☐ **Option B**

Keuzeset 12

The question for each choice set: "A 40-year-old patient has been diagnosed with stage III breast cancer with homologous recombination deficiency. Two treatments with different outcomes are possible. Which option would you choose for this patient based on the information below?"

**Attributes**	**Option A: High-dose chemotherapy with stem cell rescue (16 weeks)**	**Optie B: Standard-dose chemotherapy plus olaparib (72 weeks)**
**10-year survival rate after diagnosis**	50%	50%
**Side-effects during treatment**	Grade 2	Grade 2
**Long-term side-effects on cognition**	Moderate	Severe
**Chance of premature menopause**	80-100%	45-65%
**Choice**	☐ **Option A**	☐ **Option B**

Keuzeset 13

The question for each choice set: "A 40-year-old patient has been diagnosed with stage III breast cancer with homologous recombination deficiency. Two treatments with different outcomes are possible. Which option would you choose for this patient based on the information below?"

**Attributes**	**Option A: High-dose chemotherapy with stem cell rescue (16 weeks)**	**Optie B: Standard-dose chemotherapy plus olaparib (72 weeks)**
**10-year survival rate after diagnosis**	70%	70%
**Side-effects during treatment**	Grade 2	Grade 3
**Long-term side-effects on cognition**	Moderate	Moderate
**Chance of premature menopause**	80-100%	10-30%
**Choice**	☐ **Option A**	☐ **Option B**

Keuzeset 14

The question for each choice set: "A 40-year-old patient has been diagnosed with stage III breast cancer with homologous recombination deficiency. Two treatments with different outcomes are possible. Which option would you choose for this patient based on the information below?"

**Attributes**	**Option A: High-dose chemotherapy with stem cell rescue (16 weeks)**	**Optie B: Standard-dose chemotherapy plus olaparib (72 weeks)**
**10-year survival rate after diagnosis**	50%	60%
**Side-effects during treatment**	Grade 2	Grade 4
**Long-term side-effects on cognition**	Severe	Mild
**Chance of premature menopause**	80-100%	80-100%
**Choice**	☐ **Option A**	☐ **Option B**

Keuzeset 15

The question for each choice set: "A 40-year-old patient has been diagnosed with stage III breast cancer with homologous recombination deficiency. Two treatments with different outcomes are possible. Which option would you choose for this patient based on the information below?"

**Attributes**	**Option A: High-dose chemotherapy with stem cell rescue (16 weeks)**	**Optie B: Standard-dose chemotherapy plus olaparib (72 weeks)**
**10-year survival rate after diagnosis**	70%	50%
**Side-effects during treatment**	Grade 4	Grade 3
**Long-term side-effects on cognition**	Severe	Severe
**Chance of premature menopause**	80-100%	80-100%
**Choice**	☐ **Option A**	☐ **Option B**

PART 3: REMAINING QUESTIONS

8. In choosing the most important attributes for in the choice sets, we used survival rate, side effects during treatment, side effects on cognition, and premature menopause. Are you missing any other important attributes in the choice sets? ....

9. How would you (currently) rate the status of high-dose chemotherapy with autologous stem cell transplantation (HDCT) for stage III breast cancer patients with homologous recombination deficiency?

   ☐ Experimental

   ☐ Insufficient evidence

   ☐ Sufficient evidence

   ☐ Other, namely..

10. What are you basing your assessment of HDCT on (currently)? Multiple answers possible

    ☐ Literature

    ☐ Field of profession

    ☐ Prominent figures

    ☐ Experience

    ☐ Other, namely..

11. Have you been educated on HDCT?

    ☐ Yes

    ☐ No

12. Have you performed HDCT as a registered, ultimately responsible internist/surgeon?

    ☐ Yes

    ☐ No

    ☐ Not applicable

Finally, some statements about the application of new treatments in general (click on the circle of your choice and press 'x' on your keyboard):

13. I administer a treatment immediately if there is evidence on its effectiveness, even if colleagues (in other centers) have doubts

Totally disagree ○ ○ ○ ○ ○ Totally agree

14. I only administer a new treatment when colleagues (in other centers) have had good experiences with it

Totally disagree ○ ○ ○ ○ ○ Totally agree

15. I trust on the opinion about new treatments from prominent figures in my field

Totally disagree ○ ○ ○ ○ ○ Totally agree

CLOSING REMARKS

May we contact you if there are any uncertainties about the completed questionnaire?

☐ No

☐ Yes, you can contact me on: ………………………………………………….

Would you like to receive a summary of the results?

☐ Yes, I would like to receive a summary

☐ Nee, I don’t need to receive a summary

You can enter any comments about the questionnaire here:

………

………

………

………

We would like to thank you again for your cooperation!

Reference list

1. Vliek, S. B., Jager, A., Jongen-Lavrencic, M., Kroep, J. R., Gort, E. H., Tjan-Heijen, V. C. G., Konings, I. R., Kuip, E. J. M., Wymenga, A. N. M., Schröder, C. P., & Linn, S. C. (2018). SUBITO trial: can we improve the cure rate of stage III breast cancer with BRCA function loss substantially using intensified alkylating chemotherapy with autologous stem cell transplantation? *Nederlands Tijdschrift Voor Oncologie, 15,* 316–320.

2. Robson, M. E., Tung, N., Conte, P., Im, S. A., Senkus, E., Xu, B., ... & Wu, W. (2019). OlympiAD final overall survival and tolerability results: Olaparib versus chemotherapy treatment of physician's choice in patients with a germline BRCA mutation and HER2-negative metastatic breast cancer. *Annals of Oncology, 30*(4), 558-566.

3. National Cancer Institute,National Institutes of Health, US Department of Health and Human Services. Common Terminology Criteria for Adverse Events (CTCAE), Version 4.0. NIH publication 09-7473. Published May 29, 2009; Revised June 14, 2010. http://evs.nci.nih.gov/ftp1/CTCAE/CTCAE _4.03_2010-06-14_QuickReference_5x7.pdf. Accessed May 12, 202.

4. Blumenfeld, Z. (2012). Chemotherapy and fertility. *Best Practice & Research Clinical Obstetrics & Gynaecology*, *26*(3), 379-390

Appendix C. Distribution of choices per choice set among healthcare providers.

	HDCT	Standard of Care
Questionnaire version A (n=11 respondents)		
Choice set 1	1	10
Choice set 2	1	10
Choice set 3	1	10
Choice set 4	7	4
Choice set 5	0	11
Choice set 6	11	0
Choice set 7	11	0
Choice set 8	0	11
Choice set 9	8	3
Choice set 10	8	3
Choice set 11	11	0
Choice set 12	11	0
Choice set 13	2	9
Choice set 14	0	11
Choice set 15	11	0
Questionnaire version B (n=24 respondents)		
Choice set 16	16	8
Choice set 17	24	0
Choice set 18	8	16
Choice set 19	9	15
Choice set 20	18	6
Choice set 21	1	23
Choice set 22	2	22
Choice set 23	20	4
Choice set 24	6	18
Choice set 25	24	0
Choice set 26	1	23
Choice set 27	1	23
Choice set 28	21	3
Choice set 29	0	24
Choice set 30	2	22
